# A Medical Utopia

**Published:** 1923-06

**Authors:** 


### "A MEDICAL UTOPIA."

DR. J. WALTER CARR, consulting physician to the Royal Free Hospital, giving the annual oration of the Medical Society of London, drew a picture of what to expect in what he described as "a medical Utopia."

FROM CRADLE TO CREMATORIUM.

He pictured England under the despotic rule of the Ministry of Health, exercising a benevolent autocracy, keeping its grip on the citizen from the ante-natal period till the time when, at a ripe old age, his remains were hygienically disposed of in the municipal crematorium. The young couple about to marry would have undergone a complete medical examination, and both would be provided with certificates of fitness for matrimony. The expectant mother would be received in the municipal maternity hospital, and would be required to take her infant every week to the infant clinic until the child reached school age, and was handed over to the school medical officer. Before he reached school age, however, it would be necessary to consider what superfluous parts of the organs it was desirable to remove. The appendix would be taken out as a matter of routine, and Dr. Carr suggested that mankind would benefit by the complete removal of the entire colon, since they had the highest surgical and other authority for stating that owing to man's most unfortunate assumption of the erect posture the large intestine tended to become responsible for many of the most serious and fatal diseases to which man was liable. Operations would also be made on the tonsils, and one of the questions of the future would be whether it would not be far better to extract the second teeth at or soon after their first appearance rather than have to remove them one by one later on.

BETTER FREE THAN HEALTHY.

The greatest danger in a medical autocracy, he concluded, would be that of loss of freedom. He believed they would rather see England free than perfectly healthy. A medical despotism would be no exception to any other. It was not sufficient answer to say that medical government would be necessarily in the best interests of the governed.

### A PROBLEM OF THE TROPICS.

EVERY physician who is being trained in tropical lands spends much time and labour in acquiring a knowledge of sleeping sickness, plague, Kala-azar, Beri-beri, and the rest. Yet, when he gets to the field of his future operations, how often does he find that his main problem is only tuberculosis after all? The study of tropical tuberculosis is, to say the least of it, amazingly neglected. We do not, here at home, associate malaria and tubercle—the two seem utterly distinct. Yet overseas diagnosis between them may be immensely difficult, and life-saving depends entirely upon its correctness. Neglect to study the vagaries of tropical tuberculosis is often a paradox in an otherwise excellent organisation by which problems of tropical settlement are attacked. It is not colour, insect-borne disease, or climate which is the greatest stumbling block. Often it is just tuberculosis.

